# Body Size Measurements and Physical Performance of Youth Female Judo Athletes with Differing Menarcheal Status

**DOI:** 10.3390/ijerph182312829

**Published:** 2021-12-05

**Authors:** Marina Saldanha da Silva Athayde, Rafael Lima Kons, David Hideyoshi Fukuda, Daniele Detanico

**Affiliations:** 1 Laboratory, Center of Sports, Federal University of Santa Catarina, Florianópolis 88040, SC, Brazil; rafakons0310@gmail.com (R.L.K.); danieledetanico@gmail.com (D.D.); 2 Institute of Exercise Physiology and Rehabilitation Science, University of Central Florida, Orlando, FL 32816, USA; David.Fukuda@ucf.edu

**Keywords:** somatic maturity, puberty, combat sports, physical performance, young athletes

## Abstract

*Purpose:* To compare body size measurements and physical performance among female youth judo athletes with differing menarcheal status and to identify indicators of physical performance in post-menarcheal girls. *Methods:* Nineteen young female judo athletes (age 13.9 ± 2.3 years) were divided into a pre-menarche (*n* = 7) and a post-menarche (*n* = 12) group. The athletes were evaluated through neuromuscular tests, including standing long jump (SLJ), medicine ball throw (MBT), and handgrip strength (HGS), and judo-specific assessments, including the Special Judo Fitness Test (SJFT) and the Judogi Grip Strength Test (JGST_ISO_). Furthermore, years of experience in judo and the age at menarche were determined. *Results:* The main results showed higher performance for the post-menarche group for most variables (*p* < 0.05) compared to the pre-menarche group. A multiple linear regression analysis demonstrated that age at menarche, chronological age, and body mass explained close to 70% of JGST_ISO_, while training experience, chronological age, and age at menarche explained close to 59% of SLJ. Additionally, chronological age and age at menarche explained 40% of MBT, and chronological age and height explained 52% of HGS. *Conclusions:* Age at menarche and somatic growth variables explained moderate proportions of the variance of physical performance, thereby providing evidence that these parameters are the primary indicators of physical performance in young female judo athletes.

## 1. Introduction

Adolescence corresponds to the transition period between childhood and adulthood, during which several important biological manifestations occur, such as peak height velocity (PHV), peak weight velocity, sexual maturation, and, specifically for girls, menarche [1]. The range of variability in somatic and biological maturation among individuals of the same chronological age is large and is especially pronounced in adolescents [2]. When considering girls during this period, there is increased production of the estrogen hormone, responsible for stimulating growth and breast development [1], which is usually related to the first menstrual period (menarche) following PHV and considered an indication of biological maturation [3].

The current literature contains several studies on the effects of somatic maturity and growth on physical performance in young male athletes from team sports [4,5,6,7]. Most recently, studies have investigated the role of growth and maturity status on physical performance in young male judo athletes [8,9,10,11,12]. Years of formal judo training, growth, and somatic maturity can predict physical performance, when generalized upper and lower limb strength assessments (e.g., medicine ball throw test, handgrip strength, and jump tests) [8,10] and judo-specific tests (e.g., Special Judo Fitness Test and Judogi Grip Strength Test) [8] are considered. Moreover, Giudicelli et al. [11] found a positive relationship between maturity status and handgrip strength test as well as aerobic performance in young male judo athletes, even when chronological age and body mass were controlled. Thus, bearing in mind that judo athletes demand high levels of strength-related performance in the upper and lower limbs and both the aerobic and the anaerobic energy pathways [13], it is essential for coaches to understand the role of maturation during adolescence.

Many young female athletes are now involved in high-level judo competitions. For example, 223 female judo athletes from all continents participated in the 2019 World Cadet Judo Championship (under 18, U18), and 223 in the 2019 World Junior Judo Championship (U21) [14]. However, no studies investigating the effects of maturation on physical performance specifically in young female judo athletes have been conducted. It is known that girls present different somatic and physiological characteristics than boys due to variations in the timing and tempo of the maturation process [3,15] and, consequently, performance-related characteristics. A recent study with basketball players verified that girls with late maturity status (measured by the onset of menarche) tended to have less experience in the sport [16]. This study also found that body mass and adiposity were the highest predictors for all basketball performance indicators.

In individual sports, such as rhythmic gymnastics, Camargo et al. [17] found that there was an increase in the body fat percentage, fat mass, and fat-free mass 2 years after PHV and the occurrence of menarche. Furthermore, Pinto et al. [18] verified that girls in more advanced maturation stages presented higher values of growth indicators (weight and height) and power output of the upper limbs (through the medicine ball test). However, in individual sports of an intermittent nature requiring high-intensity actions, such as judo, these aspects are not yet fully known, in particular in relation to the physical demands of judo athletes, such as generic and judo-specific assessments.

Understanding the role of biological maturity (e.g., menarche), somatic growth measures (e.g., body size), and training experience in physical performance and their contribution in young female judo athletes can help coaches to design appropriate and individual training programs with consideration of biological development during adolescence. Thus, the purpose of this study was to compare body size measurements and physical performance among youth female judo athletes at different menarcheal statuses (pre- and post-menarche) in addition to identifying indicators of physical performance in post-menarche girls. We hypothesized that post-menarcheal girls are advanced in the growth process and consequently present higher physical performance.

## 2. Methods

### 2.1. Participants

Nineteen young female judo athletes (age 13.9 ± 2.3 years, range 10.9–17.0 years), purple (*n* = 10) and brown belts (*n* = 9), were divided into two groups: pre-menarche (*n* = 7) and post-menarche (*n* = 12). The athletes who participated in the study were from southern Brazil (Santa Catarina) and primarily of Portuguese ethnicity. The sample size was determined a priori using the GPower 3.1 software, taking as references a probability of 0.05 (minimum error type I), statistical power of 0.8 (minimum error type II), and effect size of 0.5 (mean effect). Thus, the minimum sample size was 21 participants. However, we were able to evaluate 19 athletes representing 48.7% of female athletes who participated in the competition in 2021, considering the age range of this study in the local federation (*n* = 39 athletes).

All athletes trained regularly with technical–tactical training occurring 2–3 times per week during the evaluation period, competed at the state and/or national levels, and had been engaged in formal training for at least 2 years. The girls reported no musculoskeletal disorders or injuries that would influence their maximal physical performance during the assessments. They were in the preparatory phase and therefore not in a period of rapid weight loss. All participants and responsible parties (parents and coaches/trainers) received a detailed verbal explanation of the purpose, methods, and potential risks/benefits of the study, followed by the completion of a written informed consent form. This study was approved by the Research Ethics Committee of the local university, in accordance with the Declaration of Helsinki.

### 2.2. Design

The athletes’ assessments were performed during two afternoon testing visits separated by 48 h. During the first testing visit, an interview to determine the years of judo experience and age at menarche, anthropometric measurements (body mass and height), and neuromuscular tests (standing long jump test, medicine ball throw test, and handgrip strength test) were conducted. The physical tests were separated by 20 min intervals. At the second testing visit, the young judo athletes were submitted to sport-specific tests, including the Special Judo Fitness Test (SJFT) and the Judogi Grip Isometric Strength Test (JGST_ISO_), separated by a 30 min recovery interval.

### 2.3. Determination of Menarcheal Status, Chronological Age, and Training Experience

Chronological age was calculated to the nearest 0.1 year by subtracting the date of birth from the date of testing. The number of years of formal training in judo was self-reported by the girls and/or their parents. Age at menarche was obtained through an individual interview with the girls by a female researcher; 12 athletes were determined to be post-menarche and 7 athletes pre-menarche. The body mass was measured using a digital scale Toledo^®^ (0.1 kg accuracy), and height was assessed using a stadiometer scale of 0.1 cm accuracy.

## 3. Generic Tests

### 3.1. Standing Long Jump Test

For the standing long jump test, we followed the protocol used by Detanico et al. [8]. The girls performed the standing long jump test starting from a standing position by swinging their arms and flexing their knees to provide maximal forward drive. Before the assessment, the participants performed a familiarization/warm-up of 5 min of jogging, followed by 30 s of hopping, and 5 submaximal standing long jumps. A take-off line was drawn on the ground, and the measurement of the jump length was determined using a metric tape measure (Lufkin, L716MAGCME; Apex Group, Sparks, MA, USA) from the take-off line to the nearest point of landing contact (i.e., the back of the heels). Each athlete performed three jump attempts with 2 min intervals, and the longest distance was considered for further analysis.

### 3.2. Medicine Ball Throw Test

The procedures adopted for the medicine ball throw test assessment followed the protocol of Vossen et al. [19]. The warm-up and familiarization consisted of two–three submaximal throws prior to the test. The girls remained seated on the floor covered with judo mats and were stabilized with their backs supported against a vertical support, their thighs horizontally supported, their knees flexed at an angle of 90°, and their ankles resting on the floor. A 3 Kg medicine ball (Dynamax Inc^®^., Dallas, TX, USA) was positioned at the sternum of each athlete (point A), who then threw it with both hands without moving her trunk. When an athlete failed to maintain the established body orientation, the throw attempt was disregarded. The distance the medicine ball was thrown from point A up to its first contact with the floor (point B) was measured. Each participant performed three throws, with 2 min intervals, and the greatest distance achieved in the three attempts was considered for further analysis.

### 3.3. Handgrip Strength Test

Handgrip strength was measured using a handgrip dynamometer (Carci^®^, SH 5001 model), following the protocol used by Detanico et al. [8]. The warm-up and familiarization consisted of three rapid contractions performed during a 2 s period. The participants were instructed to sustain a maximal isometric contraction during each measurement (lasting 3 to 6 s). The three contractions were performed with the dominant (self-selected) hand with 2 min intervals, in the standing position with shoulder flexion at 90° and the elbow completely extended. The highest value obtained in the three trials was considered for further analysis.

### 3.4. Judo-Specific Tests

#### 3.4.1. Special Judo Fitness Test

The SJFT is a judo high-intensity intermittent test developed by Sterkowicz [20]. The girls performed a 5 min warm-up before the test, which consisted of jogging, judo falling techniques (*ukemi*), and repetitive throwing techniques (*uchi-komi*). Subsequently, three athletes of similar body mass (with a maximum variation of 10%) and height performed the SJFT, according to the following protocol: two judokas were positioned at a distance of 6 m from each other, while the athlete being tested was positioned 3 m from the judokas to be thrown. The procedure was divided into three periods: 15 s (A), 30 s (B), and 30 s (C), with a 10 s interval between periods. In each period, the athlete being tested threw the other judoka using the one-arm shoulder throw (*ippon-seoi-nage*) technique as many times as possible. Performance was determined by the total throws completed during each of the three periods (SJFT_TT_ = A + B + C). Heart rate (HR) was measured immediately after the test and then 1 min later by an HR monitor positioned on the chest (Polar^®^ M430—Kempele/Finland). The SJFT_INDEX_ was calculated as the change in heart rate (immediately after the test and 1 min later) divided by SJFT_TT_. Previous study showed reliability values (Intraclass Correlation Coefficient—ICC) ranging from 0.71 to 0.81 for number of throws, heart rate (0.66–0.86), and SJFT index (0.87) [21].

#### 3.4.2. Judogi Grip Strength Test

The girls were familiarized with the JGST by performing one sustained attempt of 2–3 s grasping a judo uniform (*judogi*) suspended on an elevated horizontal bar. The JGST consisted of sustaining a predefined position of elbow flexion for a maximum time. Athletes performed only the isometric version of the JGST (JGST_ISO_). The chronometer began with a verbal command and was stopped when the participants could no longer maintain the original position. The reliability of the JGST has been assessed in a previous study, presenting an ICC higher than 0.98 [22].

## 4. Statistical Analysis

Data are reported as means and standard deviation (SD). The Shapiro–Wilk test was used to verify the normality of the data. Independent t-tests were used to compare the variables among girls at different menarcheal status. For the t-test, we used the Cohen’s d, considering 0.0–0.2 as trivial, 0.21–0.6 as small, 0.61–1.2 as moderate, 1.21–2.0 as large, and 2.1–4.0 as very large [23]. Multiple linear regression analysis (backward stepwise method with criteria for entry of *p* < 0.05 and removal of *p* < 0.10) was used to estimate the relative contributions of age at menarche, chronological age, years of formal training, and growth measurements (height and body mass) to physical performance. All independent variables showed variance inflation factors <2, reflecting no multicollinearity, tolerance >0.1, showing acceptable multicollinearity, and absolute values of correlation coefficients <0.70 [24]. The level of significance was set at 5%, and the analyses were performed using the JASP software (version 0.11.1, University of Amsterdam, Amsterdam, The Netherlands).

## 5. Results

Table 1 shows the demographic characteristics, judo experience, body size, and physical performance of female judo athletes of different menarcheal status. It was verified that post-menarche girls were older, more experienced, taller, and heavier and presented higher performance in SJFT (throws and index) and SLJ than pre-menarche girls. The age at menarche ranged from 10 to 13 years.

Table 2 summarizes the indicators of the physical performance tests in post-menarcheal female judo athletes. The age at menarche and body mass (negative predictors) and the chronological age (positive predictor) explained 70% of the variance in JGST_ISO_ performance. Judo training experience and chronological age (positive predictors) and age at menarche (negative predictor) explained 59% of the SLJ performance. Chronological age (positive predictor) and age at menarche (negative predictor) accounted for 40% of the variance in MBT performance, while chronological age and height (both positive predictors) explained 52% of the variance in HGS. For SJFT_TT_, no predictive analysis was reported because no variable was entered in the model using the stepwise criteria.

## 6. Discussion

The results of this study showed that post-menarche youth female judo athletes with advanced somatic growth presented higher performance in a judo-specific test (SJFT) and greater lower limb power output (SLJ) than their pre-menarche counterparts. Chronological age was an indicator in all physical tests, while age at menarche was an indicator for three of the four examined variables in post-menarche girls, thereby demonstrating that age-related maturity has an impact on general neuromuscular and judo-specific physical performance.

Similar to reports in team sports, post-menarcheal female judo athletes self-reported reaching their menarche close to 12 years. For example, Böhme [25] found the mean age at menarche to be close to 12.8 years in athletics, basketball, football, and handball athletes. Menarcheal status is an indicator of sexual maturity and usually occurs from 11 to 15 years of age, following a growth spurt (i.e., PHV) [3,26] due to hormonal alterations [1,27]. From this developmental period, advancement of motor skills and physical performance is expected, especially if there is adequate involvement in physical and sports activities from an early age [28,29]. In this study, it was verified that post-menarche youth female athletes initiated practicing judo earlier than the pre-menarche group, which may be related to their higher physical performance.

It was also found that youth female judo athletes in the post-menarche group were older, taller, and heavier than those in the pre-menarche group. Generally, adolescent girls start their growth spurt quickly, increasing approximately 8–9 cm in height per year [1] and gaining approximately 2.3–2.7 kg of body mass annually [30]. Therefore, the higher chronological age and the advanced somatic growth processes (represented by body size measurements) of post-menarche girls may explain their higher physical performance, particularly, the number of throws in SJFT and SLJ performance. A previous study verified that the number of throws in the SJFT was positively correlated with vertical jump performance in adult male judo athletes [31]. Thus, higher levels of muscle power in the lower limbs of the post-menarche girls may help to explain their higher performance in SJFT. In addition, the greater time of formal training in post-menarche girls most likely contributed to increased muscle power, as Zaggelidis et al. [32] verified that judo training enhances vertical jump performance, mainly due to improvements in the stretch–shortening cycle (SSC).

Adolescent athletes with better aerobic function present higher performance in high-intensity intermittent efforts [33]. The ability of children to better maintain performance during repeated high-intensity exercise bouts could be related to a better optimization of oxidative pathways than of glycolytic pathways during exercise and to a lower activation of type II muscle fibers, resulting in greater resistance to fatigue [34]. Although aerobic fitness was not evaluated in this study, it is possible to suggest that post-menarche girls present a higher aerobic condition than pre-menarche girls, especially due to the previously reported correlation between SJFT performance and aerobic capacity [31]. In some neuromuscular tests (HGS, MBT, JGST), there were no significant differences between pre- and post-menarche girls.

When specific indicators of physical tests were investigated in post-menarche youth judo athletes, the age at menarche was found to be negatively associated with JGST_ISO_, SLJ, and MBT (i.e., the earlier the age at menarche, the higher the performance). The status of menarche represents a great gain in the release of progesterone, estrogen, and, to a lesser extent, testosterone [35,36]. The release of estrogen and testosterone is linked to increased muscle mass and body fat, which have positive and negative influences on physical performance, respectively. In addition, the release of these hormones has been related to increases in lactic anaerobic power [37] and maximum aerobic power due to the growth of body dimensions [38].

Chronological age was a positive indicator of all neuromuscular tests, potentially demonstrating a major influence on physical performance tests in post-menarche youth female judo athletes. Detanico et al. [8] previously verified that chronological age was a positive indicator of judo-specific performance (JGST_ISO_ and SJFT variables) in young male judo athletes. Similarly, a study conducted by Courel-Ibanez et al. [39] detected a higher number of throws in the SJFT in U15 male amateur judo athletes compared to U13 athletes, showing better performance in older boys, especially when utilizing anaerobic pathways. Giudicelli et al. [12] also found that older male judokas (aged 11.0–14.7 years) performed better in most of the physical tests; however, in their study, the maturation attenuated the age effect in most variables and significantly affected upper body strength.

Another interesting finding was that the number of years of formal training was positively associated with SLJ performance, showing improvements in muscle power of the lower limbs in post-menarche girls with judo training experience. A previous investigation found that vertical jump performance discriminated adult judo athletes with different training experience levels (advanced vs. novice) [40], likely due to SSC enhancements [32]. Moreover, muscle power of the lower limbs is an important parameter related to technical–tactical performance during judo matches in senior female athletes [41].

Somatic growth variables (height and body mass) were positive and negative indicators of HGS and JGST_ISO_, respectively. Taller girls obtained advantages in the HGS test, probably due to the longer forearm and arm [1,42], which may be related to increased force production capacity. This finding exhibits practical relevance, since gripping tasks are an important component of judo performance [43]. Concurrently, body mass showed a negative impact on JGST_ISO_ performance. This test estimates isometric endurance strength in the upper body [22], and to perform it, the athletes must hold onto a bar and suspend themselves (i.e., hold their own body mass). The JGST has been previously shown to have a negative relationship with body mass [44], suggesting that heavier athletes may underperform in absolute terms in this specific test.

Finally, some limitations of this study should be addressed, such as the small sample size, particularly after division into groups according to the menarcheal status. However, the statistical power calculated a posteriori presented values >0.8 for the variables that showed significant differences, thus avoiding type II error. Nonetheless, this is the first investigation examining the effects of menarcheal status and growth on physical performance in young female judo athletes. The current results expose differences in physical performance according to the menarcheal status, showing that post-menarche girls are stronger and perform better than pre-menarche counterparts. This highlights the importance of having pre-menarche girls compete in their own age category and not in higher age groups. Age at menarche, chronological age, growth, and years of formal judo training seemed to explain the performance in post-menarche female athletes. These indicators seem to contribute to competitive success, as it was found that years of formal training, height, and strength tests performance (JGST_ISO_ and SLJ) can discriminate the competitive level in young male judo athletes (national vs. state level) [9]. However, to prevent exposure to early specialization, it is essential to consider the maturation characteristics of female youth judo athletes and individualize training loads during short- and medium-term planning. These actions will contribute to avoid harmful effects of early specialization on physical and mental health during childhood and adolescence. We recommend for future studies to investigate the influence of ethnicity, population size, parents’ education, socio-economic and nutritional parameters, as it is known that age at menarche is a sensitive indicator of environmental conditions during childhood.

## 7. Conclusions

We concluded that post-menarcheal youth female judo athletes are older, more advanced in the growth process, more experienced in judo, and present higher physical performance when compared to girls who have not yet reached menarche. Chronological age and the age at menarche were shown to be the greatest indicators of neuromuscular and judo-specific performance in post-menarche youth female judo athletes. Furthermore, somatic growth and years of formal training also contributed to neuromuscular performance of the upper and lower limbs.

## Figures and Tables

**Table 1:** Mean ± SD of demographic characteristics, judo experience, body size, and physical performance in female judo athletes of different menarcheal status.

	Pre-Menarche (*n* = 7)	Post-Menarche (*n* = 12)	*p*	ES
Chronological age (years)	11.5 ± 0.9	15.4 ± 1.5	<0.01 *	2.98
Age at menarche (years)	--	11.8 ± 1.2	--	--
Training experience (years)	4.0 ± 2.2	6.4 ± 2.5	0.03 *	1.01
Body mass (kg)	45.3 ± 3.4	59.0 ± 11.3	<0.01 *	1.36
Height (cm)	150.8 ± 5.2	159.3 ± 6.2	<0.01 *	1.47
SJFT_TT_ (*n*)	20 ± 1	23 ± 3	0.01 *	1.13
SJFT_HR_FINAL_ (bpm)	181 ± 4	183 ± 8	0.34	0.28
SJFT_HR_1MIN_ (bpm)	160 ± 6	154 ± 14	0.16	0.49
SJFT_INDEX_	16.7 ± 1.1	14.9 ± 1.9	0.02 *	1.08
JGST_ISO_ (s)	33.7 ± 9.8	43.9 ± 15.7	0.07	0.74
SLJ (cm)	177 ± 15	213 ± 45	0.04 *	0.90
MBT (cm)	277 ± 19	309 ± 71	0.15	0.50
HGS (kgf)	29.7 ± 6.9	34.8 ± 7.6	0.08	0.70

Note: * *p* < 0.05, SJFT: Special Judo Fitness Test, SJFT_TT_: total throws of SJFT, SJFT_HR_: heart rate during SJFT, JGST_ISO_: Judogi Grip Strength Test, SLJ: Standing Long Jump Test, MBT: Medicine Ball Throw Test, HGS: Handgrip Strength Test.

**Table 2:** Indicators of the physical performance tests in young post-menarcheal female judo athletes.

	Adjusted R^2^	*p*	Indicator	Standardized Coefficients (β)	*p*
JGST_ISO_ (s)	0.70	<0.01	Age-menarche	−0.788	<0.01
			Chronological age	0.783	<0.01
			Body mass	−0.803	<0.01
SLJ (cm)	0.59	0.01	Training	0.476	0.05
			Chronological age	0.526	0.04
			Age-menarche	−0.382	0.10
MBT (cm)	0.40	0.04	Chronological age	0.731	0.01
			Age-menarche	−0.449	0.10
HGS (kgf)	0.52	0.01	Chronological age	0.585	0.02
			Height	0.446	0.06

Note: JGST_ISO_: Judogi Grip Strength Test, SLJ: Standing Long Jump Test, MBT: Medicine Ball Throw Test, HGS: Handgrip Strength Test.

## Data Availability

The data are not publicly available for ethical privacy reasons with the subjects involved in the research.
